# Cost–benefit of IAPT Norway and effects on work-related outcomes and health care utilization: results from a randomized controlled trial using registry-based data

**DOI:** 10.1017/S003329172500025X

**Published:** 2025-03-13

**Authors:** Otto R.F. Smith, David M. Clark, Gunnel Hensing, Richard Layard, Marit Knapstad

**Affiliations:** 1 Department of Health Promotion, Norwegian Institute of Public Health, Bergen, Norway; 2 Centre for Evaluation of Public Health Measures, Norwegian Institute of Public Health, Norway; 3 Department of Teacher Education, NLA University College, Bergen, Norway; 4 Department of Experimental Psychology, University of Oxford, Oxford, UK; 5 School of Public Health and Community Medicine, Institute of Medicine, Sahlgrenska Academy, University of Gothenburg, Gothenburg, Sweden; 6 Centre for Economic Performance, London School of Economics, London, UK

**Keywords:** anxiety, cost–benefit, depression, health care utilization, IAPT, income, prompt mental health care, work-related outcomes

## Abstract

**Background:**

Data from an RCT of IAPT Norway (“Prompt Mental Health Care” [PMHC]) were linked to several administrative registers up to five years following the intervention. The aims were to (1) examine the effects of PMHC compared to treatment-as-usual (TAU) on work-related outcomes and health care use, (2) estimate the cost–benefit of PMHC, and (3) examine whether clinical outcomes at six-month follow-up explained the effects of PMHC on work−/cost–benefit-related outcomes.

**Methods:**

RCTs with parallel assignment were conducted at two PMHC sites (N = 738) during 2016/2017. Eligible participants were considered for admission due to anxiety and/or depression. We used Bayesian estimation with 90% credibility intervals (CI) and posterior probabilities (PP) of effects in favor of PMHC. Primary outcome years were 2018–2022. The cost–benefit analysis estimated the overall economic gain expressed in terms of a benefit–cost ratio and the differences in overall public sector spending.

**Results:**

The PMHC group was more likely than the TAU group to be in regular work without receiving welfare benefits in 2019–2022 (1.27 ≤ OR ≤ 1.43). Some evidence was found that the PMHC group spent less on health care. The benefit–cost ratio in terms of economic gain relative to intervention costs was estimated at 5.26 (90%CI 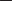 1.28, 11.8). The PP of PMHC being cost-beneficial for the economy as a whole was 85.9%. The estimated difference in public sector spending was small. PMHC effects on work participation and cost–benefit were largely explained by PMHC effects on mental health.

**Conclusions:**

The results support the societal economic benefit of investing in IAPT-like services.

## Introduction

Depression and anxiety are among the leading causes of disease burden worldwide (“Global, regional, and national burden of 12 mental disorders in 204 countries and territories, 1990–2013;2019: A systematic analysis for the Global Burden of Disease Study 2019,” 2022). Their deliberating impact on functioning and wellbeing, paired with high prevalence, often early adulthood onset, and recurrent presentations (‘Global, regional, and national burden of 12 mental disorders in 204 countries and territories, 1990–2013;2019: A systematic analysis for the Global Burden of Disease Study 2019’, 2022; Kessler et al., 2007), results not only in suffering for the individual, but also in massive costs at the family, workplace, and society levels (Kinge et al., 2017). In Norway, mental disorders are estimated to be the costliest medical condition, with a total estimated economic loss of close to 3% of GDP in 2013, relating to both healthcare expenditures and production loss such as sickness absence and disability pension (Kinge et al., 2017). Despite this, a large proportion of individuals with mental health problems, including anxiety and depression, do not receive adequate care (Alonso et al., 2018; Thornicroft et al., 2017). Scaling-up of effective prevention and treatment programs is thus regarded as essential to reduce the burden of mental health conditions (‘Global, regional, and national burden of 12 mental disorders in 204 countries and territories, 1990–2013;2019: A systematic analysis for the Global Burden of Disease Study 2019’, 2022).

The English ‘NHS Talking Therapies for Anxiety and Depression’ programme (formerly known as IAPT) represents one of the largest initiatives to increase access to evidence-based care for depression and anxiety, through a substantial investment in training new therapists and implementing a stepped-care model of empirically supported treatments nationwide (Clark, 2018; Clark et al., 2018). Inspired by its impressive results and scalability, similar models are now being rolled out in several other countries (Cano-Vindel et al., 2022; Cromarty et al., 2016; Ontario Health, 2023; Smith et al., 2017), including the ‘Prompt Mental Health Care’ (PMHC) program in Norway (Firth et al., 2015). Research have shown that both the IAPT and PMHC treatments are associated with substantial improvement in mental health symptoms and functioning (Clark, 2018; Wakefield et al., 2020). Results from a randomized controlled trial (RCT) of PMHC compared to treatment as usual (TAU) have indicated moderate to large treatment effects on symptoms, (reliable) recovery rate, functioning, and health-related quality of life at six months follow-up (Knapstad et al., 2020a). These intervention effects were maintained at 12-month follow-up (Myrtveit Sæther et al., 2020) and among participants in the PMHC group at 24- and 36-months follow-up (Smith et al., 2022). A substantial increase is also observed in self-reported work-participation (Knapstad et al., 2020b; Smith et al., 2022).

A key argument for the viability and scalability of IAPT is that it will pay for itself through gains achieved by increased work participation, tax receipts, and reduced healthcare utilization (Layard et al., 2006; Layard et al., 2007). Return on investment analyses provide a strong case for investing in treatment for anxiety and depression (Chisholm et al., 2016). However, these are partly based on modeling and assumptions that have not been fully tested. Efficacy trials of psychological interventions for depression outside of IAPT-like services show that reduction in symptoms does not necessarily translate to increased work participation. According to a recent Cochrane review, there is only low-certainty evidence that such interventions may reduce sick leave days compared to usual care among employees with depression (Nieuwenhuijsen et al., 2020). Combined clinical and work-directed interventions yield more robust findings regarding sick leave days (Nieuwenhuijsen et al., 2020). High-quality studies reporting long-term effects on functioning and occupational outcomes are scarce and called for in the literature (Kennedy et al., 2007; Nieuwenhuijsen et al., 2020; Ormel & Emmelkamp, 2023). Long-term effects may particularly be valuable for such outcomes, as they may lag symptom improvement (Kennedy et al., 2007; Smith et al., 2023) and a broad array of outcomes are needed considering both productivity and total amount of received welfare benefits.

The original return on investment case for the English IAPT programme (Layard et al., 2007) was based on research prior to the 2008 national launch of the program. Based on that research, and several key assumptions, it was argued that savings resulting from improvements in employment and reductions in other healthcare costs would more than cover the cost (£750) of providing treatment, assuming 50% of treated patients recover. The 50% recovery target was achieved in 2017 and has been maintained every year since (NHS Digital (2017–2023)). The cost of a course of treatment is also broadly in line with expectation (Clark, 2018). However, few studies have linked datasets to assess in practice the savings that were anticipated and those that have assessed savings have tended to focus only a component of the IAPT programme and/or on a subset of potential savings. A study (Gruber et al., 2022) that compared IAPT treated patients with depression or anxiety who also had a long-term physical health problem with a control population who had not received IAPT treatment, reported substantial savings in hospital inpatient and out-patient costs. Another linkage study (El Baou et al., 2023) followed patients up for three years after treatment in IAPT. Compared to a propensity matched control group, depressed patients who recovered with IAPT treatment had a reduced rate of new cardiovascular events (including strokes and heart attacks) that required medical attention during follow-up. A further study (Toffolutti et al., 2021) used a stepped-wedge implementation design to assess the local impact of starting a new IAPT service that specially focused on people with depression/anxiety in the context of long-term health problems. Compared to untreated controls in the late implementation areas, treated patients in the early implementation areas showed substantial savings in hospital inpatient and outpatient costs in the first three months. They also reported an overall eight percentage point higher employment rate during the following year. Finally, a comparison (Department of Work and Pensions, 2022) between out-of-work IAPT treated patients who also received the support of an employment advisor, (as recommended in the IAPT service model), with a propensity-matched control group who did not receive such additional support, found that addition of the employment advisor was associated with a greater increase in rates of employment. While encouraging, these studies each only assess a subset of possible savings, and none use a gold standard design in which patients are randomly allocated to IAPT or the control condition.

In sum, while there is solid evidence of the clinical effectiveness of IAPT, its overall cost – benefit and effects on work-related outcomes and healthcare use are less clear. In the current study, data from the PMHC trial were linked to several administrative registers, which provided us with near complete outcome data up to five years following the intervention. The aims were: (1) to examine the effects of PMHC compared to treatment as usual (TAU) on work-related outcomes and health care use, (2) to estimate the benefit to cost ratio of PMHC, and (3) to examine whether intervention effects on work participation and cost – benefit were explained by intervention effects on self-reported clinical outcomes at six-month follow-up (i.e., symptoms).

## Methods

Design, study setting, recruitment, randomization, and interventions were described in detail in an earlier article that presented the main clinical findings of the trial (Knapstad et al., 2020a). Key aspects will be summarized below.

The trial was conducted within routine care at, and in close collaboration with, two PMHC sites; Kristiansand and Sandnes. To be eligible for PMHC service during the trial period, the patient had to present with anxiety and/or mild to moderate depression as determined by the PMHC staff during initial assessment. A randomized controlled design with parallel assignment was used. The participants were randomized (using a computerized random number generator) on a 70:30 ratio (PMHC versus TAU) with simple randomization within each of the two sites and with no further constraints. A 70:30 ratio was used to make the PMHC program available to as many clients as possible while at the same time ensuring a control group of sufficient size. PMHC is a commitment to give more people an evidence-based form of CBT appropriate for their clinical condition, using people properly trained to do so. CBT treatment is offered in both low-intensity (guided self-help, psycho-educational groups) and high intensity (individual cognitive-behavior therapy) formats. The care is organized according to a type of matched-care model, in which information from the initial assessment and patient preferences is used to determine the choice of treatment. The provided treatments utilize multiple specific CBT protocols that target both depression and a wide range of anxiety disorders, which are theoretically anchored in models such as the Clark and Wells treatment model for social anxiety disorder, the Clark and Salkovski treatment model for panic disorder, the Barlow and Craske model for panic disorder, the Borkovec model for generalized anxiety disorder, the Wells metacognitive model for rumination and worry, and the Beck depression model. Treatment as usual included all ordinary services available to the target population. In Sandnes and Kristiansand, as in many Norwegian municipalities, this usually involves clinical follow-up and intervention by the GP but can also include assistance from private practice psychologists and occupational health services, or no treatment at all.

### Ethical consent and trial registration

The trial was reported according to the CONSORT statement and is registered at ClinicalTrials.gov (NCT03238872). No changes to the design were made after trial commencement. The trial protocol was approved by the Regional Ethics Committee for Western Norway (REK-vest no. 2015/885) [21, 23].

### Data collection

**Figure 1.**
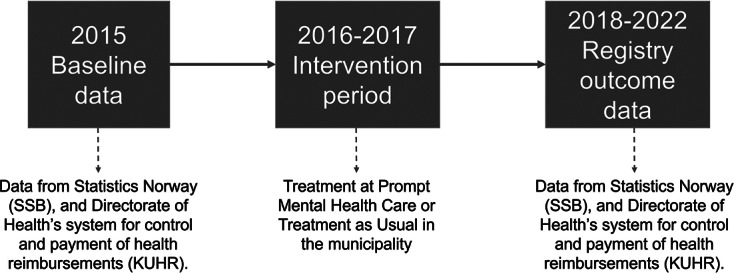
Information on data collection and study phases.

Registry data was obtained for years 2015–2022. As the intervention was primarily taking place in 2016 and 2017, the years 2018–2022 were considered the primary outcome years. Data from 2015 was treated as a baseline covariate (see Figure 1). Self-reported data on symptoms of anxiety and depression was obtained at baseline and 6-month follow-up.

Annual occupational income and annual welfare benefit data were derived from Statistics Norway (SSB) and were available for years 2015–2022. Data on healthcare utilization were derived from the Directorate of Health’s system for control and payment of health reimbursements (KUHR). This information was available for years 2015–2019 (years 2020–2022 were not available when we applied for the data). The registry contains individual-level information on bills from the majority of publicly funded health services, including outpatient specialist health care. The KUHR registry does not include costs associated with inpatient health care and fully private health care expenditures. All included registries are regarded as highly accurate and complete.

### Participant flow

Between November 9, 2015 and August 31, 2017, 527 (68.1%) patients were randomized to the PMHC condition and 247 (31.9%) patients were randomized to the TAU condition. A total of 22 participants did not consent to registry linkage, whereas it was not possible to carry out the registry linkage for an additional 14 participants due to ID entry errors. Registry data were available for 738 participants, which represents 95.3% of the original sample: 508 (96.4%) in the PMHC group and 230 (93.1%) in the TAU group. Self-reported symptom outcome data at six-month follow-up was available for 62.4% of the participants in the PMHC group (*n* = 170), and 45.0% of the participants in the TAU group (*n* = 98). Participants who were retired or on permanent disability leave at baseline were not included in the analyses considering the work-related outcomes (*n* = 22) as they were no longer part of the workforce.

### Implementation and fidelity evaluation

As previously reported (Knapstad et al., 2020a; Lervik et al., 2020), PMHC was largely implemented as intended during this RCT. The services seemed to reach the intended target group, waiting times were relatively short (27 days, IQR 18–39), there was no waiting list, and self-referral was used relatively often (33%).

The PMHC group received a mean of 6.3 (SD = 4.5) treatment sessions and 76.9% completed treatment (therapist reporting that treatment goal was fulfilled and/or completed at least six sessions), and patients reported high treatment satisfaction. Group-based psychoeducation was the primary treatment form for 35.1%, individual CBT for 30.0%, and guided self-help for 0.9%. The remaining 34.0% received a combination of these treatment forms. A fidelity evaluation based on routinely audio-recorded sessions found the CBT treatment delivered to be within sufficient range. Notably, however, the integrated work focus in treatment was found to be relatively low, as was collaboration with other services, such as GPs, patients’ workplaces, or social services (Lervik et al., 2020).

In the TAU group, of those responding to the six -month follow-up questionnaire, 58.7% reported having received health care for their mental health problems since baseline. Most reported follow-up by the GP and/or by a psychologist/psychiatrist (Knapstad et al., 2020a).

### Outcome variables

Our primary outcome aimed to resemble being in regular work without receiving welfare benefits, like our previous studies based on self-reported work outcomes (Knapstad et al., 2020a; Knapstad et al., 2020b; Myrtveit Sæther et al., 2020; Smith et al., 2022). In the present study, being in regular work was defined as earning an occupational income of at least 50% of the average Norwegian income during a given year. As data on hours employed were not available, we used this as a proxy for working in a 50% position or more, which is the case for over 90% of the Norwegian workforce (Statistics Norway, 2013). Without receiving welfare benefits was defined as not having received sick leave benefits (i.e., ≥14 days. Sick leave up to 14 days is covered by the employer and not recorded), work assessment allowance benefits, disability pension benefits, social welfare and/or unemployment benefits during a given year. Throughout the article, we refer to this binary outcome variable as employed without receiving welfare benefits (yes/no).

Secondary work outcomes included yearly occupational income (in 1000 NOK), being a recipient of welfare benefits in a given year (yes/no), number of consultations in public healthcare services for all causes in a given year, and number of consultations in public health care services for mental health-related causes in a given year.

### Mediator variables

Symptoms of depression were measured using the Patient Health Questionnaire (PHQ-9), which assesses the frequency of nine symptoms (“not at all” [0] to “nearly every day” [3]) in the last two weeks [25, 26]. A total score ranging from 0 to 27 was created. The PHQ-9 has been shown to have good psychometric properties [25] and the Cronbach’s alpha was 0.80 in our sample. Symptoms of anxiety were measured using the General Anxiety Disorder-7 (GAD-7), comprising seven items with similar frequency ratings and time frame to the PHQ-9 [26, 27]. A total score ranging from 0 to 21 was created. GAD has displayed good reliability and validity for measuring generalized anxiety disorder [27] and satisfactory sensitivity and specificity for generalized anxiety and other anxiety disorders [28]. In our sample, the Cronbach’s alpha for GAD-7 was 0.83. For the sake of simplicity, we operationalized symptoms of anxiety and depression as a single latent variable (i.e., a total score alternative that accounts for measurement error), as supported by a study from Kroenke et al. (Kroenke et al., 2016).

### Cost – benefit analysis

A societal perspective was used for the cost – benefit analyses in which the effect of the intervention on the economy at large and its effects on public sector spending was assessed. Intervention costs were based on reports from the participating municipalities for the years 2016 and 2017 and included both clinician’s wages and service operating costs. Costs for PMHC therapist training and education were based on information obtained from the Norwegian Association for Cognitive Therapy. Costs of publicly funded health care utilization were derived from KUHR. Data from Statistics Norway (SSB) was used to obtain yearly received welfare benefits (sick leave benefits, disability pension benefits, unemployment benefits, and work assessment allowance (WAA), and yearly gross occupational income.

### Statistical analyses

Mplus version 8.8 was used for all analyses. Linear regression was used for continuous outcomes, logistic regression for binary outcomes, and negative binomial regression for count outcomes. The following covariates were included in all models: baseline variable of the outcome variable for the year 2015 (i.e., prior to randomization) and site (Sandnes, Kristiansand). All models were based on Bayesian estimation and used the default non-informative priors. 90% credibility intervals were used in this study, which is in line with recommendations from earlier work (Kruschke, 2014). The probability of an effect in favor of PMHC was computed based on the posterior distribution.

For the cost – benefit analyses, we considered both the effect of the intervention on the economy at large and its effects on public sector spending. For the former, the total earned occupational income minus total healthcare spending was used as a measure of the overall economic gain and calculated for each participant for the period post-randomization to the end of 2022. The average time from post-randomization to the end of 2022 was 6.3 years. The outcome was regressed on the group variable (intervention vs. control), site, and the baseline version of the outcome variable in the year prior to randomization. The estimated effect was then divided by the average investment cost per patient of the PMHC intervention to obtain the benefit – cost ratio (BCR) at the societal level. Welfare benefits were not included in this regard as these were considered transfer payments (Treasury Kaitohutohu Kaupapa Rawa, 2015).

Public sector spending was calculated for each participant as the total received welfare benefits (SSB) and state-covered health care spending (KUHR) for the period post-randomization to the end of 2022. As health care costs were not available for 2020–2022, the last observed value from 2019 was carried forward. The cost of the PMHC intervention was added to participants who received PMHC treatment. The outcome of public sector spending was also regressed on the group variable (intervention vs. control), site, and the baseline version of the outcome variable in the year prior to randomization. Costs and benefits were discounted at 4% per year. Sensitivity analyses were conducted at discount rates of 0% and 7% per year (Appendix A).

**Figure 2.**
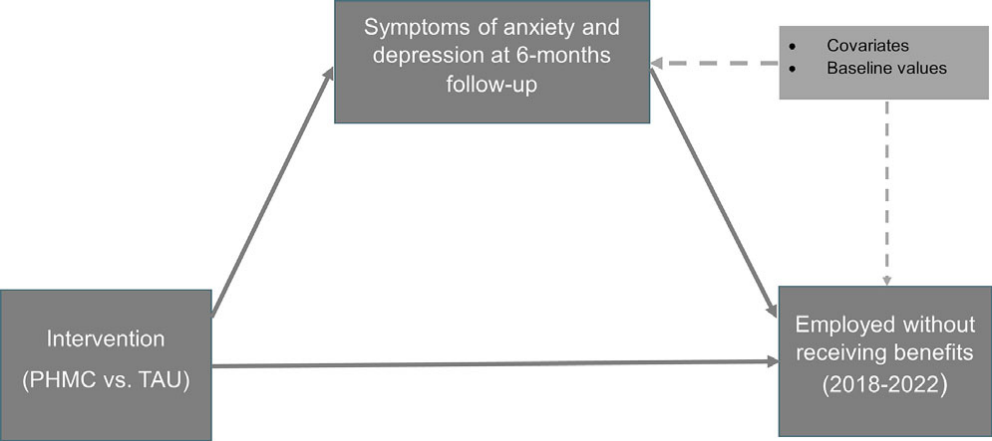
Path diagram of the tested mediation model.

Finally, we examined the extent to which the intervention effects on the primary outcome, being employed without receiving welfare benefits in 2018–2022, were explained by the intervention effect on symptoms of anxiety and depression at the six-month follow-up. That is, two regression equations were estimated simultaneously in Mplus. The first one with the outcome as the dependent variable and symptoms, intervention group, and covariates as independent variables. The second one with symptoms as the outcome and intervention group and covariates as independent variables. The regression estimates were used to derive the indirect effect of the intervention on regular work via symptoms and were calculated separately for each outcome year. The same covariates were included as in the outcome analyses with the addition of the baseline values for symptoms of anxiety and depression (see also Figure 2). Mediation analysis was also carried out for overall economic gain as the outcome variable.

## Results

### Descriptive statistics

As displayed in Table 1, baseline characteristics were generally similar across the two treatment groups. In total, two-thirds of the participants were women, and the mean age was 34.8 (SD = 12.1) years. Mean severity scores of PHQ-9 = 14.0 (SD = 5.0) and GAD-7 = 11.2 (SD = 4.6) were within the expected range of the target group. In 2015, 35.8% of the sample was employed without receiving any welfare benefits that year, suggesting that many of the participants were (partly) on welfare benefits (46.6%). The remaining 17,6% were out of the workforce (full time students, homemakers, disability pensioners, retirees). The mean occupational income in 2015 was 327,145 NOK (SD = 264,580), which can be considered low according to Norwegian standards (540,500 NOK in 2015), but this can partly be explained by the fact that not all people in our sample were part of the work force.

**Table 1.** Baseline characteristics by the treatment group

Variable	Type	PMHC group (N = 508)	TAU group (N = 230)	Total (N = 738)
Age	mean (SD)	34.8 (11.9)	34.8 (12.6)	34.8 (12.1)
Female sex	% (*n*)	65.4 (332)	67.4 (155)	66.0 (487)
PHQ baseline	mean (SD)	13.9 (5.0)	14.0 (5.0)	14.0 (5.0)
GAD baseline	mean (SD)	11.3 (4.6)	11.1 (4.6)	11.2 (4.6)
Employed and no welfare benefits in 2015^†^	% (*n*)	31.4 (154)	31.6 (71)	31.5 (225)
Occupational income 2015^*^ ^†^	mean (SD)	328.4 (268.0)	324.5 (257.5)	327.1 (264.6)
Recipient of welfare benefits 2015^†^	% (*n*)	47.1 (231)	45.3 (102)	46.6 (333)
Health care consultations, all-cause 2015	mean (SD)	5.8 (3.4)	5.5 (3.3)	5.7 (3.4)
Health care consultations, mental cause 2015	mean (SD)	1.3 (2.2)	1.2 (2.2)	1.3 (2.2)

* x 1000 NOK

† N = 715.

### Primary outcome

**Figure 3.**
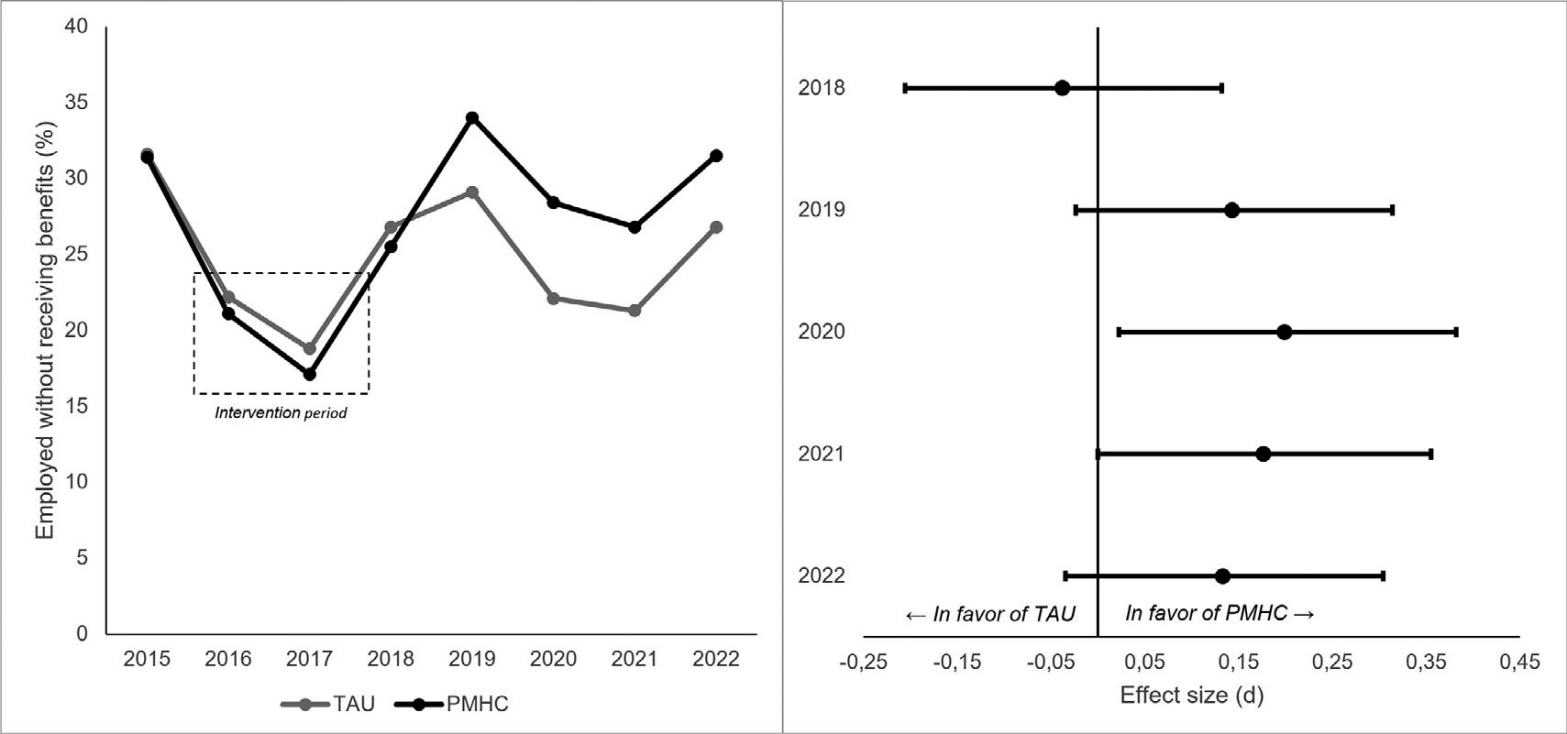
Being employed without receiving benefits by year and group (left) and effect size estimates for years 2018–2022 (right). *Note:* the standardized effect size was calculated by applying the following formula: *d = In(OR) × (* 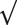 *3/π*).

As shown in Table 2, participants assigned to the PMHC group were more likely to be employed while not receiving welfare benefits in years 2019 to 2022 as compared to TAU. The posterior probabilities of an effect in favor of PMHC ranged from 90.6 to 96.9%. There was no indication of an intervention effect in 2018. As expected, the standardized effect sizes were small with point estimates ranging from −.04 to .20 (see also Figure 3).

**Table 2.** Intervention effects on work-related outcomes and healthcare utilization

	Descriptive statistics				Intervention effect	
Outcome	Type	Intervention	Control		Estimate (90% CI^a^)	Posterior probability of effect in favor of PMHC
Employed and no welfare benefits 2018	% (*n*)	25.5 (123)	26.8 (60)		OR = .93 (.69, 1.27)	35.0%
Employed and no welfare benefits 2019	% (*n*)	34.0 (164)	29.1 (65)		OR = 1.30 (.96, 1.77)	92.1%
Employed and no welfare benefits 2020	% (*n*)	28.4 (136)	22.1 (49)		OR = 1.43 (1.04, 2.00)	96.9%
Employed and no welfare benefits 2021	% (*n*)	26.8 (128)	21.3 (47)		OR = 1.38 (1.00, 1.90)	95.0%
Employed and no welfare benefits 2022	% (*n*)	31.5 (150)	26.8 (59)		OR = 1.27 (.94, 1.74)	90.4%
Occupational income 2018 (x 1000 NOK)	mean (SD)	324.8 (268.0)	321.9 (258.7)		B = 1.4 (−23.0, 25.8)	53.7%
Occupational income 2019 (x 1000 NOK)	mean (SD)	360.9 (294.8)	337.5 (270.8)		B = 21.8 (−7.0, 50.6)	89.6%
Occupational income 2020 (x 1000 NOK)	mean (SD)	369.3 (298.2)	333.1 (266.8)		B = 33.8 (4.9, 63.4)	97.4%
Occupational income 2021 (x 1000 NOK)	mean (SD)	389.5 (308.9)	354.1 (285.8)		B = 31.2 (−1.1, 64.5)	94.2%
Occupational income 2022 (x 1000 NOK)	mean (SD)	415.7 (329.0)	384.0 (318.7)		B = 27.4 (−8.6, 64.1)	89.4%
Recipient of welfare benefits 2018^b^	% (*n*)	61.0 (294)	55.8 (125)		OR = 1.14 (.96, 1.36)	10.6%
Recipient of welfare benefits 2019^b^	% (*n*)	53.9 (260)	54.7 (122)		OR = .96 (.96, 1.36)	64.4%
Recipient of welfare benefits 2020^b^	% (*n*)	64.9 (311)	69.8 (155)		OR = .85 (.71, 1.02)	92.9%
Recipient of welfare benefits 2021^b^	% (*n*)	69.6 (332)	71.9 (159)		OR = .92 (.77, 1.10)	77.5%
Recipient of welfare benefits 2022^b^	% (*n*)	63.5 (311)	64.5 (142)		OR = 1.01 (.85, 1.20)	47.3%
Number of healthcare consultations 2018 All causes Mental health-related causes	mean (SD) mean (SD)	6.5 (3.5) 2.2 (3.2)	6.9 (3.7) 2.4 (3.4)		IRR = .93 (.87, 1.01) IRR = .90 (.70, 1.12)	93.7% 76.7%
Number of healthcare consultations 2019 All causes Mental health related causes	mean (SD) mean (SD)	6.4 (3.7) 2.1 (3.2)	6.6 (3.6) 2.2 (3.2)		IRR = .96 (.89, 1.03) IRR = .94 (.75, 1.16)	83.6% 69.3%

B, Unstandardized regression coefficient; OR, Odds ratio; IRR, Incidence Risk Ratio.

a Credibility interval.

b Sick leave benefits work assessment allowance, unemployment benefits, social welfare benefits, and/or disability pension.

### Secondary outcomes

**Figure 4.**
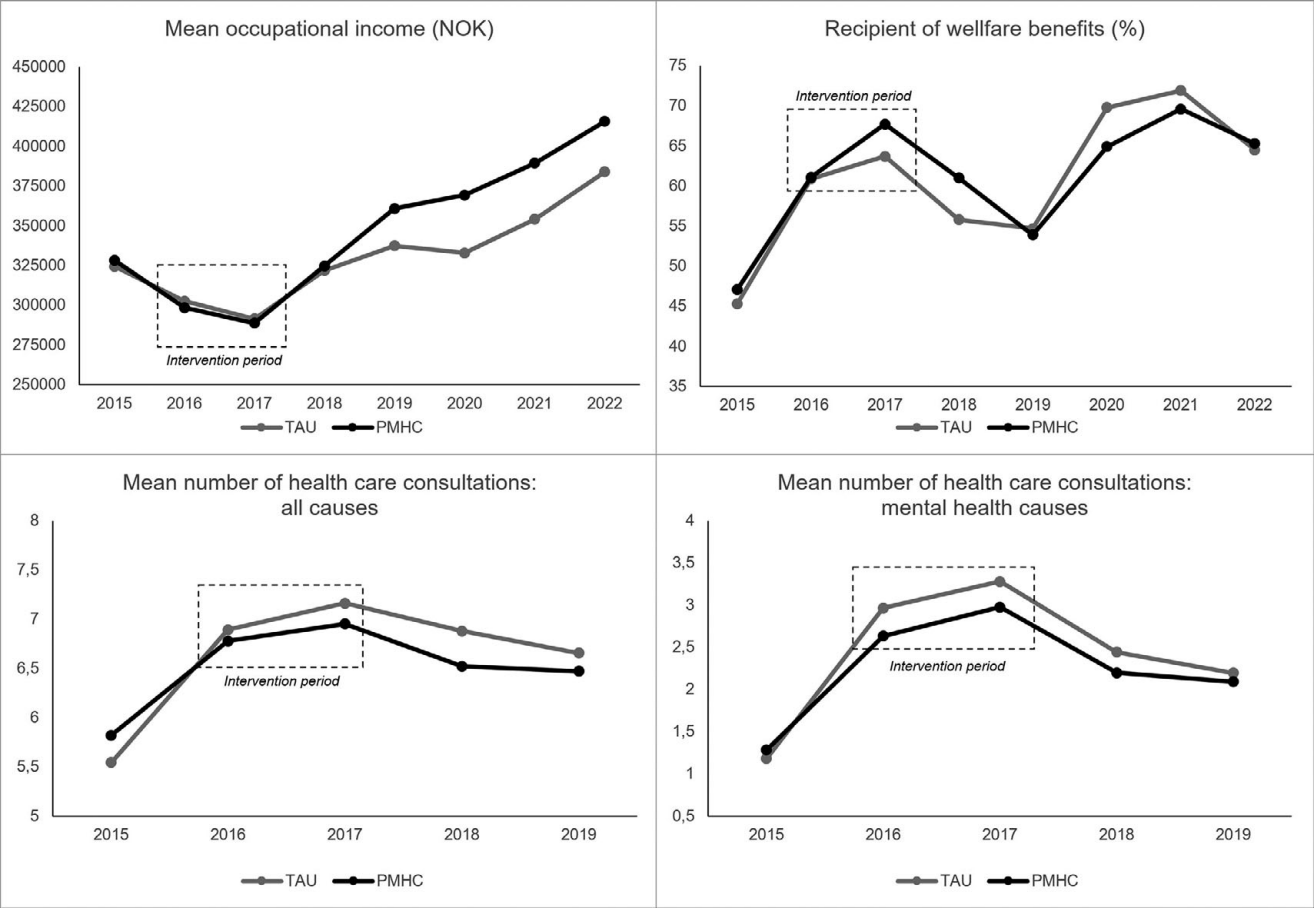
Secondary outcomes by year and group.

For the outcome of annual occupational income, the pattern was quite similar to that of the primary outcome (Table 2). That is, the intervention effect was more in favor of PMHC in years 2019–2022 and absent in 2018. For being a recipient of welfare benefits, results were more in favor of TAU in 2018, similar for both groups in 2019 and 2022, and more in favor of PMHC in 2020 and 2021, but the differences were small overall. We also estimated the intervention effects for the two most common types of welfare benefits in this sample (sick leave benefits and work assessment allowance), but no clear pattern was found (see Appendix B). Finally, some evidence was found that participants in the PMHC group had fewer health care service consultations in 2018 and 2019 as compared to TAU, in particular for all causes and to a lesser degree for mental health-related causes. The posterior probability of an effect on healthcare utilization in favor of PMHC varied between 69.3% and 93.7%. Overall, the observed patterns indicated intervention effects in favor of PMHC (see Figure 4).

### Cost – benefit analyses

**Figure 5.**
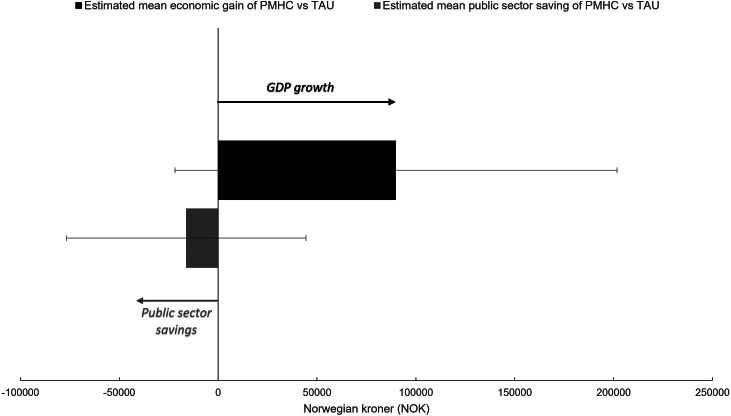
Estimated effects of PMHC vs. TAU on economic gain and public sector spending from post-randomization to year 2022.

Based on the data provided by the participating municipalities and the Norwegian Association for Cognitive Therapy, the estimated average cost per PMHC patient was 17,054 NOK. Results from the regression analysis indicated that the average economic gain in favor of PMHC was 89,680 NOK (90%CI 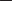 21,850 NOK, 201,710 NOK) across the entire follow-up period. Dividing this estimate by the average cost of the intervention resulted in a benefit – cost ratio of 5.26 (90%CI 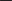 1.28, 11.8, PP = 91%). The posterior probability of a benefit – cost ratio greater than one was 85.9%. No clear evidence was found for a difference in overall public sector spending between the PMHC and TAU groups. Based on the available data, public sector spending was estimated to be 16,050 NOK lower on average in the PMHC group (90% CI −76,760 NOK, 44,450 NOK). The posterior probability of PMHC being associated with public sector savings compared to the treatment-as-usual group was 67%. Overall, the results were suggestive of the PMHC intervention having a positive effect on the economy at large at no additional costs to the public sector (see also Figure 5).

### Mediation analyses

Table 3 shows the results for the total, direct, and indirect effects of the mediation model displayed in figure 2. The effect of the PMHC intervention on being employed without receiving welfare benefits was to a substantial degree mediated by the effect of the intervention on symptoms at six-month follow-up for outcome years 2019–2022. The percentage of the total effect explained by the mediator ranged from 47 to 77%.

**Table 3.** Total, indirect, and direct effects of the mediation model in which the PMHC effect on selected outcomes is mediated by its effects on symptoms

Outcome	Year	Total effect	Indirect effect	Direct effect	% explained
Primary outcome^a^	2018	−.06 (−.38, .26)	.14 (−.00, .32)	−.21 (−.56, .14)	-
	2019	.28 (−.04, .60)	.21 (.06, .39)	.06 (−.29, .41)	75%
	2020	.38 (.05, .71)	.18 (.02, .36)	.20 (−.16, .56)	47%
	2021	.35 (.02, .68)	.17 (.01, .36)	.17 (−.20, .54)	49%
	2022	.26 (−.05, .58)	.20 (.04, .39)	.06 (−.29, .40)	77%
Overall economic gain	All years^b^	8.82 (−2.32, 19.90)	9.64 (4.33, 16.13)	−1.03 (−13.42, 11.17)	≈100%

a Employed without receiving welfare benefits.

b From post-randomization to end of 2022.

A similar mediation analysis was conducted with the overall economic gain as an outcome. The results from this analysis indicated that the effect of the intervention on overall economic gain was largely explained by the intervention effect on symptoms at six-month follow-up (Table 3).

## Discussion

This is the first study that examined the long-term economic impact of an IAPT-like service based on an RCT design, with objective and near complete outcomes up to five years follow-up. The trial had a high participation rate (only 3.3% of the eligible declined) and registry data was available for 95.3% of the original sample. Overall, the observed patterns were suggestive of intervention effects in favor of PMHC. Participants in the PMHC group were more likely to be employed without receiving welfare benefits in years 2019–2022, while this effect was more uncertain in 2018. Similar patterns were observed for occupational income and to a lesser extent for being a recipient of welfare benefits. Moreover, some evidence was found for effects on health care spending. The benefit–cost ratio in terms of economic gain relative to intervention costs was estimated at 5.26, while the estimated differences in public sector spending between the intervention and control groups were minimal. This suggests that the PMHC program had little cost to the public sector and very positive benefits to the economy as a whole. Finally, mediation analyses suggested that the intervention effects on work participation and cost – benefit were to a large degree explained by the intervention effects on mental health at six-months follow-up.

The results on work-related outcomes expand the evidence base both within the IAPT/PMHC context (Knapstad et al., 2020a; Myrtveit Sæther et al., 2020) and in the field more generally, where findings are mixed and knowledge on long-term effects limited (Nieuwenhuijsen et al., 2020; Nigatu et al., 2016). We found positive effects on employment in years 2019–2022 but not in 2018. The latter is in contrast to some studies in English and Spanish IAPT (Department of Work and Pensions, 2022; Munoz-Navarro et al., 2024; Toffolutti et al., 2021), where effects were observed in the immediate post-treatment year. PMHC as implemented during the trial had a relatively low degree of work focus in treatment (Lervik et al., 2020), which may partly explain the lack of effect in the short term. There is growing evidence that incorporating an explicit work focus in treatment may be important for achieving more rapid effects on work outcomes (Nieuwenhuijsen et al., 2020; Øverland et al., 2018). Further developing this aspect within the context of PMHC should definitely be considered as it will likely improve its impact on work outcomes, and thereby its societal impact. Many English IAPT services already include specialist employment advisors who work alongside the psychological therapists.

The increasing effects in favor of PMHC at longer-term follow-up suggests that full social and employment recovery after episodes of anxiety and depression can sometimes be a protracted process (Øyeflaten et al., 2012). The findings are also in line with a recent PMHC study that showed that functional improvement at 12-month follow-up lagged symptom change at 6-month follow-up (Smith et al., 2023). CBT provides patients with tools on how to deal with certain psychological problems, but it may take both time and effort before these techniques are fully integrated in a person’s everyday life. Once they do, it may make an individual who underwent PMHC treatment more robust for setbacks in the longer run compared to someone who did not. Methodologically, it underscores the importance of including long enough follow-up times in trials to tease out these effects. It is also worth noting that the follow-up period included years 2020–2022, thus, the outbreak of the Corona pandemic and associated lock-down measures and layoffs. The increase in total received welfare benefits in both study groups in 2020 compared to previous years (see Table 2) might reflect consequences of the pandemic. The increased effects in favor of PMHC this year are, perhaps, even more interesting in light of this unforeseen societal shock. As mentioned, one interpretation could be that the PMHC treatment provided this group with skills that better enabled them to handle such a crisis. In support of this interpretation, the mediation analyses indicated that at least half of the effect on work participation in the years 2020–2022 could be explained by intervention effects on mental health symptoms at six-months follow-up.

Another important result, both from a secondary prevention and health economic point of view, is the indication of effects on both mental and all-cause health care use. Individuals with anxiety and depression often have co-existing physical health problems, which in turn is linked to more physical health care use (Layard & Clark, 2015). The results lend support to the notion that mental health treatment may have a ripple of effects on all cause health care use, through better self-management or improvements in physical health. Few empirical studies exist on this matter, but similar findings are indicated in three previous IAPT studies among patients with a co-occurring long-term physical health condition; two of the studies observed a reduction in in-patient and outpatient hospital utilization 12 months following IAPT treatment, compared to a matched control sample (Gruber et al., 2022), and another reported reduced emergency department attendance, but not inpatient and outpatient use, using a controlled before and after design (de Lusignan et al., 2013). The effect on all-cause health care is particularly noteworthy as individuals with physical health conditions as the main problem were excluded from the current trial.

Direct comparisons to cost – benefit analyses of IAPT in England are difficult due to differences in study designs, operationalizations of costs, resource use, and welfare benefits, and not least in health and social security systems between countries. PMHC interventions are all based on cognitive behaviorbehavior therapy, whereas IAPT supports a range of NICE-recommended psychological interventions (Clark, 2018). Among low-intensity interventions, the included PMHC sites focused more on group-based psychoeducation, while IAPT generally uses more guided self-help. The estimated cost per patient (17,054 NOK) was considerably higher in PMHC than in IAPT (Clark, 2018). This is partly because the number of treated patients per therapist per year is typically lower in PMHC as compared to IAPT. Costs also need to be seen in context, where Norway for instance has higher salaries and, not least, is among the countries worldwide spending most on healthcare in general (Kinge et al., 2023). Despite these higher costs, the economic cost – benefit remained in favor of PMHC.

Anxiety and depression are among the costliest medical conditions, related to both healthcare spending (Kinge et al., 2023) and production loss (Kinge et al., 2017). One may question whether a net economic gain of 14,235 NOK per treated patient per year (89,680/6.3), as observed in the current study, is satisfactory in a health economic perspective. Scaled to annual national numbers illustrate that small gains can have a large societal, health economic impact (Chisholm et al., 2016; Layard & Clark, 2015); Even if one conservatively assumes a 50% lower real life effect size due to possible overestimation in underpowered studies (Button et al., 2013), scaled to the estimated yearly number of treated patients in PMHC today (15 000), the yearly economic gain could be close to 110 million NOK per year. Today about 60% of the adult population live in a municipality that has implemented PMHC. Thus, if available in all municipalities, yearly gains could accumulate to over 175 million NOK each year (≈16 million USD with exchange rate 1 USD = 10.5 NOK). Furthermore, the current study concentrated on societal perspective for the return in investment analysis. Future studies are warranted that endorse a personal perspective, where the value of PHMC compared to TAU are measured in terms of gains in Quality Adjusted Life Years (QALYs). As PMHC was associated with better clinical outcomes than TAU, it will be associated with an increase in QALYs. We did not include this in our financial model as we only had EQ-5D data up to 12-month follow-up. The cost-utility analysis based on the available data in this project will be published in a separate paper.

The finding that effects of the intervention on work-related and cost-effectiveness outcomes were mediated by the effects of the intervention on mental health implies that efforts to improve psychological outcomes in PMHC will lead to better societal outcomes as well. To illustrate this point, we broke down the intervention group by those who were reliably recovered versus those who were not and compared both groups to TAU. This yielded a benefit – cost ratio of 13.0 for recovered patients and a benefit – cost ratio of −1.4 for unrecovered patients, that is recovered patients were associated with much higher economic gains compared to non-recovered patients, as also suggested by a recent Spanish study (Barrio-Martinez et al., 2024). Continuous implementation efforts and investments in more therapists per PMHC site, to allow for more tailored treatment and more sessions per patient, are therefore of great importance to improve psychological and societal outcomes and are likely to pay for themselves (Clark et al., 2018; Layard & Clark, 2015). The measurement-based care model in which routinely collected outcome data is used to progressively refine and improve the effectiveness of the services is another example of how IAPT is trying to further improve its outcomes. Future studies should also consider including other potential mechanisms by which the PMHC intervention can have an impact on work-related and cost-effectiveness outcomes, such as cognitive processes and acquisition of particular skills.

When interpreting the findings, both strengths and weaknesses must be considered. The pragmatic RCT design and use of a wide range of administrative register data in the current study circumvented some limitations in previous IAPT papers and meets the general call for trials on anxiety and depression that are conducted in real-world settings and address long-term outcomes, including functional outcomes (Ormel et al., 2022). First and foremost, objective registry-based outcomes greatly reduce the risk of bias relating to attrition, recall, and reporting. Recall bias can be a particular challenge when aiming to ascertain benefits and healthcare use over a longer time period (Johns & Miraglia, 2015) and reporting bias in trials where blinding is impossible and subjective outcomes are used (Hernán, 2004; Wood et al., 2008). Registry-based data are moreover recommended in the occupational health field to facilitate the synthesis of evidence across studies (Nieuwenhuijsen et al., 2020). The included registries are being used for reimbursement of welfare benefits and are thus regarded as highly accurate and complete. Moreover, information on operating costs and therapist training was derived directly from the providers and regarded trustworthy.

Although the study sample size is relatively large for symptom-based outcomes, for work-related outcomes and cost – benefit analyses where the anticipated effect sizes are smaller, this sample size is on the small side and also below the initially planned sample size of 1108 (Knapstad et al., 2020a). That is, the design of the current study had low statistical power, and the latter is associated with inflated effects sizes and low reproducibility (Button et al., 2013). The presented results should therefore be interpreted with caution.

Despite the many advantages of registry-data, their structure does not always match the needs for particular research purposes. In this study, for example, an occupational income of at least 50% of the average Norwegian income was used as a proxy for working in a 50% position or more. The health care data retrieved include neither inpatient hospitalisation, private expenditure on psychological treatment, medical prescriptions nor follow-up in the PMHC service. This is likely associated with an underestimation of the health care costs in both study groups (Kinge et al., 2023). It is uncertain to what extent such data would change the between group effects. However, it is worth noting that hospitalization costs account for most of the savings in healthcare utilization that have been observed in English IAPT studies (de Lusignan et al., 2013; Gruber et al., 2022; Toffolutti et al., 2021).

In both PMHC and IAPT, a substantial proportion of those who are assessed for the services (30–45%) do not enter treatment (assessed non-suitable or decline treatment) (Knapstad et al., 2020a; Knapstad et al., 2018). We have not evaluated the possible societal gain (or costs) of such single-session assessments, which can involve advice, and signposting to more appropriate help, potentially relieving some of the work for GPs.

In conclusion, the results support the societal benefit of investing in IAPT-like services. Our estimates have some uncertainty because of the relatively low sample size set against the expected effect sizes of the included outcomes. Still, this study may be the best evidence available to date on this matter in the context of IAPT. The findings from the mediation analyses strengthened our confidence in the presented results, as they provide empirical evidence for the mechanism by which IAPT is expected to impact work outcomes, namely by improving mental health.

## Supporting information

Smith et al. supplementary material Smith et al. supplementary material

## Supplementary material

The supplementary material for this article can be found at http://doi.org/10.1017/S003329172500025X.
