# Supplementary material for: Cost–benefit of IAPT Norway and effects on work-related outcomes and health care utilization: results from a randomized controlled trial using registry-based data
Source: Psychol Med. 2025 Mar 13;55:e86. doi: 10.1017/S003329172500025X (PMC12080634; doi:10.1017/S003329172500025X)
Supplement: Smith et al. supplementary material [file S003329172500025Xsup001.docx]

| **Appendix A. Cost-benefit estimates based on different discount rates.** | | | |
| --- | --- | --- | --- |
|  | Estimated intervention effect  with discount rate at 0% | Estimated intervention effect  with discount rate at 4% | Estimated intervention effect  with discount rate at 7% |
| Overall economic gain (x 10,000 NOK) | 10.74 (-2.24, 23.72) | 8.97 (-2.19, 20.17) | 7.80 (-2.20, 17.86) |
| Public sector spending (x 10,000 NOK) | -1.80 (-8.67, 5.11) | -1.61 (-7.68, 4.45) | -.94 (-6.20, 4.35) |
| Benefit-to-Cost ratio (BCN) | 6.30 (-1.31, 13.91) | 5.26 (-1.28, 11.83) | 4.58 (-1.29, 10.47) |
| Posterior probability of BCN>1 | 87.4% | 85.9% | 84.4% |
|  |  |  |  |

| **Appendix B. Intervention effects on sick leave benefits and work assessment allowance.** | | | | | |  |
| --- | --- | --- | --- | --- | --- | --- |
| **Outcome (x 1000 NOK)** | **Descriptive statistics** | | |  | **Intervention effect** | |
|  | Type | Intervention | Control |  | Estimate (90% CI^1^) | Posterior probability of effect in favour of PMHC |
| Recipient of sick leave benefits 2018 | % (n) | 42.3 (204) | 37.9 (85) |  | OR = 1.11 (.94, 1.32) | 15.0% |
| Recipient of sick leave benefits 2019 | % (n) | 34.4 (166) | 33.2 (74) |  | OR = 1.03 (.86, 1.22) | 39.8% |
| Recipient of sick leave benefits 2020 | % (n) | 40.7 (195) | 42.3 (94) |  | OR = .95 (.80, 1.13) | 69.2% |
| Recipient of sick leave benefits 2021 | % (n) | 45.8 (218) | 45.2 (100) |  | OR = 1.01 (.85, 1.19) | 47.7% |
| Recipient of sick leave benefits 2022 | % (n) | 42.2 (201) | 40.0 (88) |  | OR = 1.05 (.89, 1.25) | 30.4% |
|  |  |  |  |  |  |  |
| Recipient of work assessment allowance 2018 | % (n) | 19.3 (93) | 19.2 (43) |  | OR = .96 (.79, 1.16) | 64.3% |
| Recipient of work assessment allowance 2019 | % (n) | 17.0 (82) | 17.9 (40) |  | OR = .95 (.78, 1.16) | 66.1% |
| Recipient of work assessment allowance 2020 | % (n) | 16.1 (77) | 17.6 (39) |  | OR = .92 (.76, 1.12) | 74.8% |
| Recipient of work assessment allowance 2021 | % (n) | 17.0 (81) | 18.1 (40) |  | OR = .95 (.78, 1.16) | 66.1% |
| Recipient of work assessment allowance 2022 | % (n) | 17.6 (84) | 16.8 (37) |  | OR = 1.02 (.84, 1.25) | 42.1% |
